# Editorial: Psychotic experiences and symptoms in adolescents and young adults from different countries and cultural backgrounds

**DOI:** 10.3389/fpsyt.2023.1275061

**Published:** 2023-09-04

**Authors:** Feten Fekih-Romdhane, Sahar Obeid, Souheil Hallit

**Affiliations:** ^1^Department of Psychiatry “Ibn Omrane”, The Tunisian Center of Early Intervention in Psychosis, Razi Hospital, Manouba, Tunisia; ^2^Faculty of Medicine of Tunis, Tunis El Manar University, Tunis, Tunisia; ^3^Department of Social and Education Sciences, School of Arts and Sciences, Lebanese American University, Jbeil, Lebanon; ^4^School of Medicine and Medical Sciences, Holy Spirit University of Kaslik, Jounieh, Lebanon; ^5^Department of Psychology, College of Humanities, Effat University, Jeddah, Saudi Arabia; ^6^Department of Research, Psychiatric Hospital of the Cross, Jal Eddib, Lebanon; ^7^Applied Science Research Center, Applied Science Private University, Amman, Jordan

**Keywords:** psychotic experiences, adolescents and young adults, culture, psychosis risk, early intervention in psychosis

As stipulated by the dimensional approach, the psychotic phenomena occurs in the general population through a continuum ranging from subclinical psychotic experiences (PEs) to severe, full-blown psychotic disorders (1). PEs have been shown to be associated with a propensity to develop later psychotic disorders, thus representing an extended psychosis phenotype, as well as with a broad range of subsequent psychopathology, behavioral problems, and poor functioning (2). Several previous investigations pointed to higher levels of PEs in low-and-middle-income-countries (LAMIC) than in high-income countries (HIC) (3, 4), which could, in part, be explicable by cultural differences. Indeed, cross-cultural studies showed that people tend to perceive PEs as more culturally acceptable in LAMIC (5), which likely contributes to greater likelihood of reporting PEs and lower subsequent distress. In addition, some patterns of putative causal mechanisms for PEs (such as urban living, socioeconomic disparities, cannabis use, and racial discrimination) have proven to vary widely between nations (6). The overall picture of these findings emphasizes that the prevalence of, and risk factors influencing, PEs appear to be more pronounced in people living in some countries, mirroring the well-documented inequality in health care access and quality across countries (7). Indeed, some countries are confronted with limited resources, low availability of mental health services, inequities in access to care, limited global health policy attention, and high levels of public stigma related to mental illness [e.g., (8)].

While strong efforts have been made over the last years in prevention and early intervention for psychosis in several countries, such efforts are still largely inadequate or completely lacking in others. In some parts of the world, such as the Middle East and North Africa (MENA) region, early intervention in psychosis services and research in this area are still emerging [e.g., The Tunisian Center of Early Intervention in Psychosis (TCEIP) (9)]. There have been some very recent attempts to advance early intervention in countries where the progress in this field remains slow, by investigating subclinical PEs in the general population [e.g., (3, 10)], or by translating and validating essential measurement tools in the field in local languages [e.g., the Prodromal Questionnaire-Brief in Arabic (11), the Psychosis Screening Questionnaire in sub-Saharan African languages (12)]. A recent systematic review on studies around the ultra-high risk (UHR) for psychosis paradigm emerging from developing countries could identify no studies from low-income countries, and only eight from LAMIC (13). Altogether, it is apparent that some countries remain to date slow and/or deficient in complying with international standards of early intervention. These countries face some key challenges to the development and implementation of early intervention services for young people with psychosis, including the non-recognition of early psychotic symptoms, or the non-recognition of the different biological and environmental risk factors for the development of psychosis. In countries where there has been no progress at all in the field, or only sparse coverage, prevention and early intervention programmes should be tailored to the local cultural and social context, as previously implemented programmes may not be universal and may not be applicable in every culture or country. In short, there is a need to advance knowledge of the prevalence, nature, markers and risk factors of early symptoms of psychosis in under-researched countries' populations.

Therefore, this Research Topic focuses on PEs among adolescents and young adults from the general population of different countries and cultural backgrounds, and comprises five empirical papers. Chen and Toulopoulou examined PEs in relation to lifelong school bullying experiences among Chinese adolescents and young adults, and constructed multiple mediation models to systematically investigate the mechanisms underlying this relationship. They demonstrated a direct and indirect effect of bullying intensity on PEs through self-esteem, neuroticism personality traits, and a cognitive bias in thinking called interpretation bias. The present Research Topic encompasses two Chilean studies. Chile has only recently joined the Latin American psychosis early detection programs (EDPs) initiative. In this regard, Gaspar et al. (14) have recently published the results of their first experience in establishing the “The University of Chile High-risk Intervention Program”, where they describe their cohort (*N* = 27 UHR youths) and the challenges encountered in the implementation process. The first Chilean study by Wastler and Núñez sought to explore the association between PEs, emotion regulation, and suicidal ideation among 1,590 adolescents from the general population of Chile. Findings pointed to a moderate association between PEs (i.e., paranoid ideation, perceptual abnormalities, and bizarre experiences) and suicidal ideation. In addition, greater cognitive reappraisal and expressive suppression were significantly linked to suicidal ideation. Results from the logistic regression revealed that PEs (paranoid ideation, perceptual abnormalities) and expressive suppression are strongly associated with suicidal ideation, even after adjusting for demographic variables and depression. In sum, these results corroborate those of previous studies from other developing countries that found a close link between PEs and high rates of suicidal ideation (15). Another Chilean study by Langer et al. aimed to better understand how PEs relate to various psychological processes and psychiatric symptoms (i.e., depression, suicidal behavior, anxiety, post-traumatic stress disorder, entrapment/defeat and emotional regulation) using latent profile analysis (LPA) and a sample of adolescent general population. Four distinct profiles were identified mainly based on levels of depression, posttraumatic stress symptoms and defeat/entrapment: “low symptomatology” (19.1%), “mild-moderate symptomatology” (39.4%), “moderate symptomatology” (33.7%), “high symptomatology” (7.8%). Authors concluded that when assessing mental health risk in adolescents, practitioners should be aware that PEs occurs in the context of other psychopathology and transdiagnostic affective and cognitive processes. A Qatari study by Yehya et al. used a qualitative phenomenological approach and semi-structured interviews to examine the phenomenology of PEs in a non-clinical sample of female university students. In Qatar, there are yet no early intervention services implemented, to the best of our knowledge. Findings highlighted that PEs were prevalent, commonly normalized and linked to real-life events, and shaped to some extent by culture and religion. These results provide additional support to previous findings that culture has a substantial impact on the content, frequency, and reaction to PEs (16). Jiang et al. reviewed the demographics and clinical information for Chinese children and adolescents with depression in different age groups of onset. They found that 25.0% of depressive children and adolescents had psychotic symptoms. In addition, they showed that females in the children group were more likely to have psychotic symptoms, whereas males in the late adolescence group had fewer psychotic symptoms. Authors suggested that clinicians are required to use proficient and flexible communication skills to identify psychotic symptoms in affected children, as they may be reluctant to mention their abnormal thinking and perception because of embarrassment.

## Author contributions

FF-R: Writing—original draft. SO: Writing—review and editing. SH: Writing—review and editing.
